# A multifaceted intervention to reduce guideline non-adherence among prescribing physicians in Dutch hospitals

**DOI:** 10.1007/s11096-017-0553-0

**Published:** 2017-11-03

**Authors:** Jacqueline M. Bos, Stephanie Natsch, Patricia M. L. A. van den Bemt, Johan L. W. Pot, J. Elsbeth Nagtegaal, Andre Wieringa, Gert Jan van der Wilt, Peter A. G. M. De Smet, Cornelis Kramers

**Affiliations:** 1 Department of Clinical Pharmacy, Canisius Wilhelmina Hospital, Weg door Jonkerbos 100, 6532 SZ Nijmegen, The Netherlands; 2 Department of Pharmacy, Radboud University Medical Center, Nijmegen, The Netherlands; 3 Department of Hospital Pharmacy, Erasmus University Medical Centre, Rotterdam, The Netherlands; 4 Department of Clinical Pharmacy, Meander Medical Centre, Amersfoort, The Netherlands; 5 Department of Clinical Pharmacy, Isala Hospital, Zwolle, The Netherlands; 6 Department of Health Evidence, Radboud University Medical Centre, Nijmegen, The Netherlands; 7 Department Scientific Institute for Quality of Healthcare, Radboud University Medical Centre, Nijmegen, The Netherlands; 8 Department of Clinical Pharmacology and Toxicology, Radboud University Medical Centre, Nijmegen, The Netherlands

**Keywords:** Education, Guideline adherence, Medication review, Patient safety, Prescribing, The Netherlands

## Abstract

*Background* Despite the potential of clinical practice guidelines to improve patient outcomes, adherence to guidelines by prescribers is inconsistent. *Objective* The aim of the study was to determine whether an approach of introducing an educational programme for prescribers in the hospital combined with audit and feedback by the hospital pharmacist reduces non-adherence of prescribing physicians to key pharmacotherapeutic guidelines. *Setting* This prospective intervention study with a before–after design evaluated patients at surgical, urological and orthopaedic wards. *Method* An educational program covering pain management, antithrombotics, fluid and electrolyte management, prescribing in case of renal insufficiency, application of radiographic contrast agents and surgical antibiotic prophylaxis was presented to prescribers on the participating wards. Hospital pharmacists performed medication safety consultations, combining medication review of patients who are at risk for drug related problems with visits to ward physicians. *Main outcome measure* The outcome measure was the proportion of the admissions of patients in which the physician did not adhere to one or more of the included guidelines. Difference was expressed in odds ratios (OR) with 95% confidence intervals (CI). Multivariable logistic regression analysis was performed. *Results* 1435 Admissions of 1378 patients during the usual care period and 1195 admissions of 1090 patients during the intervention period were included. Non-adherence was observed significantly less often during the intervention period [21.8% (193/886)] as compared to the usual care period [30.5% (332/1089)]. The adjusted OR was 0.61 (95% CI 0.49–0.76). *Conclusion* This study shows that education and support of the prescribing physician can reduce guideline non-adherence at surgical wards.

## Impacts on practice

- Pharmacotherapeutic guidelines seem to be poorly implemented in daily clinical practice in Dutch hospitals.
- Hospital pharmacists can play a leading role in the implementation of key pharmacotherapeutic guidelines.
- Education on guidelines and support of the prescribing physician by the hospital pharmacist are an effective way to improve guideline adherence.

## Introduction

Preventable, clinically relevant problems due to complex pharmacotherapy are common among hospitalised patients [1–4]. Examples are haemorrhage, arterial or venous thrombosis, drug intoxication in renal insufficiency, delirium and faecal impaction. Many of these problems derive from prescribing errors that lead to potentially preventable morbidity, mortality and costs [5]. The majority of these are caused by pain medication, antithrombotics, antibacterial drugs, cardiovascular drugs, and drugs that are renally excreted [1–3, 6–9].

Different strategies, including introduction of computerized physician order entry (CPOE), pharmacist involvement on the ward, educational programs and support systems for clinical decision making (CDS) have been studied to address this problem and to improve clinician prescribing in hospitalized patients [10–12].

Clinical practice guidelines with evidence-based recommendations for physicians have been developed to assist doctors and to improve patient outcomes. In routine daily practice however, it appears to be difficult to implement key recommendations and guidelines seem to have limited impact on physician prescribing behaviour. Most clinicians can barely keep pace with the rapid advances in pharmacotherapy. And even if doctors are aware of the guidelines and are willing to change, to alter well established patterns of prescribing is difficult [13]. Earlier research showed that non-compliance to several guidelines by prescribers varies between 33 and 70% [14–16].

Several determinants of practice that prevent or enable guideline adherence, have been described. Guideline factors, such as quality of evidence and accessibility of the guideline, organizational factors and resources, such as the information system, frequent rotations of physicians on the ward and workload, patient factors such as increasingly complex multi-morbidity and also individual health professional factors, such as knowledge and skills, awareness and professional behaviour play a role [17, 18]. When these factors are taken into account in the development of strategies to improve guideline adherence, the quality of the treatment of hospitalised patients improves [19, 20].

Education is one of the possible strategies to tackle several of these determinants for non-adherence. Education of prescribers is most effective when it is interactive and continuous, includes discussion of evidence and local consensus and when it is followed by feedback on performance. This way of professional development needs to be built into patient care as much as possible, and should preferably take place in real time with clinical decision-support tools and patient-specific reminders to help doctors make the best decisions [21].

The P-REVIEW study is a prospective, multicentre, open intervention study, designed to investigate if an approach of introducing an educational programme for prescribers in the hospital combined with audit and feedback by the hospital pharmacist can lead to a clinically relevant benefit for patients at surgical wards [22]. The educational program teaches the prescriber the pharmacological aspects of using high-risk drugs in high-risk patients. The hospital pharmacist suggests interventions based on a medication review of the patient. Guidelines are an important part of the educational program and the hospital pharmacist actively checks on and improves guideline adherence.

## Aim of the study

The aim of the study was to determine whether an approach of introducing an educational programme for prescribers in the hospital combined with audit and feedback by the hospital pharmacist reduces non-adherence of prescribing physicians to key pharmacotherapeutic guidelines.

## Ethics approval

The institutional review boards of the Isala Hospital (Zwolle, the Netherlands) and the Meander Medical Centre (Amersfoort, the Netherlands) stated that the study was exempt from ethical approval. Patients’ data were collected and stored in accordance with prevailing privacy regulations.

## Methods

### Study design and setting

The P-REVIEW study was an open intervention study with a before-after design performed in two large general teaching hospitals in the Netherlands (the Isala Hospital (779 beds), and the Meander Medical Centre (600 beds)) [22]. After a 6-month control period (usual care) the intervention was introduced during 3 months. This was followed by a 6-month intervention period. This sub-study on guideline adherence was performed during the fifth month of the usual care period and the fifth month of the intervention period.

### Study population

Patients who were admitted to the surgical, urological and orthopaedic wards of the two hospitals during the study period were included. Guideline non-adherence was measured in all these patients. Patients were followed up until discharge. Patients could be included more than once, in case of readmission in the study period. Day care patients were excluded.

### Usual care

During the usual care period the normal procedures of medication surveillance and communication between hospital pharmacists and physicians were maintained. A CPOE and CDS system was applied in both hospitals.

Hospital pharmacists checked medication of all patients on a daily basis with the aid of computer-generated alerts based on a national database (“G-standard”) [23]. They could warn the physician by telephone or in case the advice was less urgent send a an advice on paper to the ward. In both hospitals, pharmacists were supported by a same set of computerised “clinical rules” to screen for specific prescribing errors. These clinical rules are based on pharmacotherapeutic guidelines and combine clinical patient data (like renal function and electrolyte abnormalities) with medication specific factors: dose adjustments in case of renal insufficiency; hypokalemia in patients using diuretic; hyperkalemia in patients using potassium-saving diuretics, ACE inhibitor, trimethoprim or NSAID; hyponatremia in patients using SSRI, thiazide or carbamazepine; folic acid to be added to methotrexate; dosing of oral cytostatics; PPI to be added in case of NSAID [24].

### Intervention

During the intervention phase, a combination of an educational program and medication counselling for prescribers on the wards took place.

An educational program covering pain management, antithrombotics, fluid and electrolyte management, prescribing in the case of renal insufficiency, application of radiographic contrast agents and surgical antibiotic prophylaxis was developed. National and local hospital guidelines related to these subjects were also included [25–32]. The program consisted of two parts of approximately 2 h each. All prescribers, who provided medical care on the participating wards during the intervention period, attended the course.

In addition, hospital pharmacists were trained to perform medication safety consultations (MSC), combining medication reviews and a visit to audit and give feedback to prescribers on the ward by an internist clinical pharmacologist and a hospital pharmacist with specific expertise in this area. Medication reviews were performed in high-risk patients, who were identified with a computerised screening method. The screening method was based on recent literature on prescription errors and targeted patients at risk for potentially preventable, drug-related problems. This screening method and a checklist for performing medication review on surgical wards is described before by Bos et al. [22]. In the weekly visits of the hospital pharmacist to the physician on the surgical ward, there was special attention for adherence to important pharmacotherapeutic guidelines that were addressed in the educational program. Feedback was given based on the medication reviews to the prescriber. The attended issues and advices were discussed in a broader context and hospital pharmacists clarified the pharmacological background and related prevailing hospital guidelines.

### Guidelines

In order to be able to score guideline non-adherence ten recommended pharmacotherapeutic measures were derived from several guidelines (Table 1).

**Table 1:** Pharmacotherapeutic measures based on prevailing guidelines

Pharmacotherapeutic measure	Effectuation measurement of guideline non-adherence	Guideline
1. Perioperative thrombosis prophylaxis	All patients undergoing surgery, with a high risk of thrombosis according to the guideline, were checked whether preventive therapy for DVT and VTE was administered	Diagnostics, prevention and treatment of venous thromboembolism and secondary prevention of arterial occlusive disease (guideline CBO, based on ACCP) [25]
2. Perioperative bridging of antithrombotics	All patients undergoing surgery, using vitamin K antagonists, were checked whether perioperative bridging of antithrombotics was indicated and antithrombotics were administered according to the guideline. Bridging was indicated in case of atrial fibrillation and a CHADS_2_ score > 3, recent or recurrent venous thromboembolism, thromboembolism due to thrombophilia or mechanical valve prosthesis	Diagnostics, prevention and treatment of venous thromboembolism and secondary prevention of arterial occlusive disease (guideline CBO, based on ACCP) [25]
3. PPI added in case of use of NSAID	All patients with an ulcer in history and/or an age older than 70 years, were checked whether a proton pump inhibitor was added	NSAID use and prevention of gastric damage (guideline CBO) [26]
4. Laxative added in case of use of opioid	All patients treated with an opioid, were checked whether a laxative was added. Patients with a stoma or with diagnosed diarrhoea were excluded	Pain (guideline NHG) [27], Diagnostics and treatment of pain (guideline Oncoline) [28]
5. NSAID contraindicated in impaired renal function	All patients with an impaired renal function (MDRD < 30 ml/min/1.73 m^2^), were checked for NSAID use	Dutch national G-standard [23] SmPC NSAID [29]
6. Discontinuation of diuretics in case of radiocontrast	All patients who received iodinated radio-contrast and who used diuretics, were checked whether the diuretic was discontinued on the day of the test	Precautions for use of iodinated radio-contrast (guideline NVR) [30]
7. Discontinuation of NSAID in case of radio-contrast	All patients who received iodinated radio-contrast and who used an NSAID, were checked whether the NSAID was discontinued on the day of the test	Precautions for use of iodinated radio-contrast (guideline NVR) [30]
8. Discontinuation of metformin in case of radio-contrast and impaired renal function	All patients who received iodinated radio-contrast and had impaired renal function (MDRD < 60 ml/min/1.73 m^2^) and used metformin, were checked whether metformin was discontinued on the day of the test	Precautions for use of iodinated radio-contrast (guideline NVR) [30]
9. Perioperative antibiotics prophylaxis	All patients undergoing surgery, with an indication for perioperative antibiotics prophylaxis, were checked whether preventive therapy for infection was administered	Perioperative antibiotic prophylaxis (guideline SWAB) [31]
10. Perioperative endocarditis prophylaxis	All patients undergoing surgery, with a high risk of endocarditis, were checked whether preventive therapy for endocarditis was administered	Endocarditis prophylaxis (guideline by the Netherland Heart Foundation) [32]

*ACCP* American College of Chest Physicians, *CBO* Dutch Institute for Health Care Improvement, *DVT* deep vein thrombosis, *MDRD* modification of diet in renal disease, *NHG* Dutch Society of General Practitioners, *NSAID* non-steroidal anti-inflammatory drug, *NVR* Dutch Association of Radiology, *PPI* proton pump inhibitor, *SmPC* Summary of Product Characteristics, *SWAB* The Dutch Working Party on Antibiotic Policy, *VTE* venous thromboembolism

The guidelines were selected by a group of experts, including hospital pharmacists, clinical pharmacologists and hospital-based physicians in a consensus meeting. The selected guidelines had to relate to medication that has shown to frequently be involved in preventable, clinically relevant, drug-related problems [1–3, 6–8]. All guidelines had to be part of a local implemented protocol in the hospital and were addressed in the educational program.

### Study endpoints

The primary outcome measure of guideline non-adherence was the proportion of the admissions of patients in which the physician did not adhere to one or more of the guidelines. The secondary outcome measures were the proportions of admissions of patients in which the physician did not adhere to each of ten guidelines.

### Data collection

Collected data included patient characteristics, laboratory and medication data, as well as transfers to other wards, medical correspondence and medical interventions. Data regarding radiology, microbiology, blood transfusion and information about medical incidents were also collected. Part of the requisite data could not be collected automatically. Therefore, a trained research assistant collected data manually from the medical records of the patients using a predefined protocol. These data included whether the patient had had surgery, type of surgery and whether the patient had an indication for thrombosis prophylaxis, antibiotics prophylaxis or endocarditis prophylaxis.

A validated multisource Microsoft Access database (Microsoft version 2003) was used.

### Sample size and data analysis

The PREVIEW-study has been powered on the outcome measure of reduction of clinically relevant, potentially preventable drug-related problems. For the power of this sub-study on guideline adherence, we studied earlier research on this subject showing that non-compliance to several guidelines by prescribers varies between 33 and 70% [14–16]. Earlier studies that describe interventions that aim to improve guideline adherence showed results on improvement of adherence varying form 50–60 to 65–80% [19, 20]. To detect a reduction from 30% non-adherence to 20% non-adherence, 313 patients had to be included in each group. Because the primary outcome measure of the P-REVIEW study (adverse drug events) needed a very large patient cohort to detect a significant difference, we assumed that measuring during 1 month in both periods would generate enough power for this sub-study on guideline adherence.

Baseline characteristics were presented as means and standard deviation or percentages for continuous or dichotomous outcomes, respectively.

Differences between groups were expressed in odds ratios with 95% confidence intervals and were tested for statistical significance using independent *t* test or Chi square tests, as appropriate. P < 0.05 was considered to be statistically significant.

In order to correct for possible confounding, multivariable logistic regression analysis was performed. The following possible confounders were initially entered into the model: age, gender, department of admission, number of medicines on the first day after admission and pharmacotherapeutic group of these medicines, length of stay and renal function. Those that showed no clear relation with the outcome (*p* > 0.10) were removed, but only in case their removal did not alter the relation under study (OR on non-adherence in usual care period vs intervention period) by more than 10%.

Statistical analyses were performed using SPSS Statistics version 22 (IBM Software, New York).

## Results

In the usual care period of the study 1435 admissions (1378 patients) and in the intervention period 1195 admissions (1090 patients) were included.

Table 2 details the characteristics of these patients. There was no difference between the two groups in age, gender, department of admission or in the number of medications on the first day after admission. Also, there was no difference in use of medication, length of hospital stay and the proportion of patients with renal insufficiency.

**Table 2:** Characteristics of admitted patients

	Usual care period	Intervention period	*p* value
No. of admissions	1435	1195	
No. of patients	1378	1090	
Mean age of patients in years ± SD	63.8 ± 17.2	63.3 ± 17.1	0.406
Gender of patients, n (%) female	720 (50.2%)	599 (50.1%)	0.980
Department of admission			0.605
General surgery, n (%)	852 (59.4%)	682 (57.1%)	
Orthopaedic surgery, n (%)	328 (22.9%)	294 (24.6%)	
Urology, n (%)	255 (17.8%)	219 (18.3%)	
Mean no. of medications the first day after admission, ± SD	6.9 ± 5.5	7.2 ± 5.8	0.233
Medication the first day after admission, n (%)			
Hypoglycemics	178 (12.4%)	156 (13.1%)	0.618
Vitamin K antagonists	149 (10.4%)	117 (9.8%)	0.616
Heparin/LMWH	951 (66.3%)	773 (64.7%)	0.394
Thrombocyte aggregation inhibitors	284 (19.8%)	238 (19.9%)	0.936
Diuretics	337 (23.5%)	287 (24.0%)	0.749
Beta blockers	391 (27.2%)	305 (25.5%)	0.318
Calcium channel blockers	146 (10.2%)	142 (11.9%)	0.162
RAS inhibitors	375 (26.1%)	317 (26.5%)	0.819
NSAIDs	485 (33.8%)	424 (35.5%)	0.366
Opioids	601 (41.9%)	491 (41.1%)	0.681
Antipsychotics	90 (6.3%)	79 (6.6%)	0.724
Mean length of stay, days ± SD			
General surgery	7.7 ± 9.7	7.0 ± 8.3	0.154
Orthopaedic surgery	7.6 ± 8.6	6.7 ± 6.5	0.107
Urology	4.3 ± 4.8	4.4 ± 4.1	0.798
MDRD eGFR of patients (ml/min/1.73 m^2^), n (%)	(n = 1016*)	(n = 836*)	0.476
< 10	4 (0.4%)	1 (0.1%)	
10–30	43 (4.2%)	39 (4.7%)	
30–60	227 (22.3%)	203 (24.3%)	
> 60	742 (73.0%)	593 (70.9%)	

*LMWH* low molecular weight heparin, *RAS* renin angiotensin system, *NSAIDs* non-steroidal anti-Inflammatory drugs

**Fig. 1:**
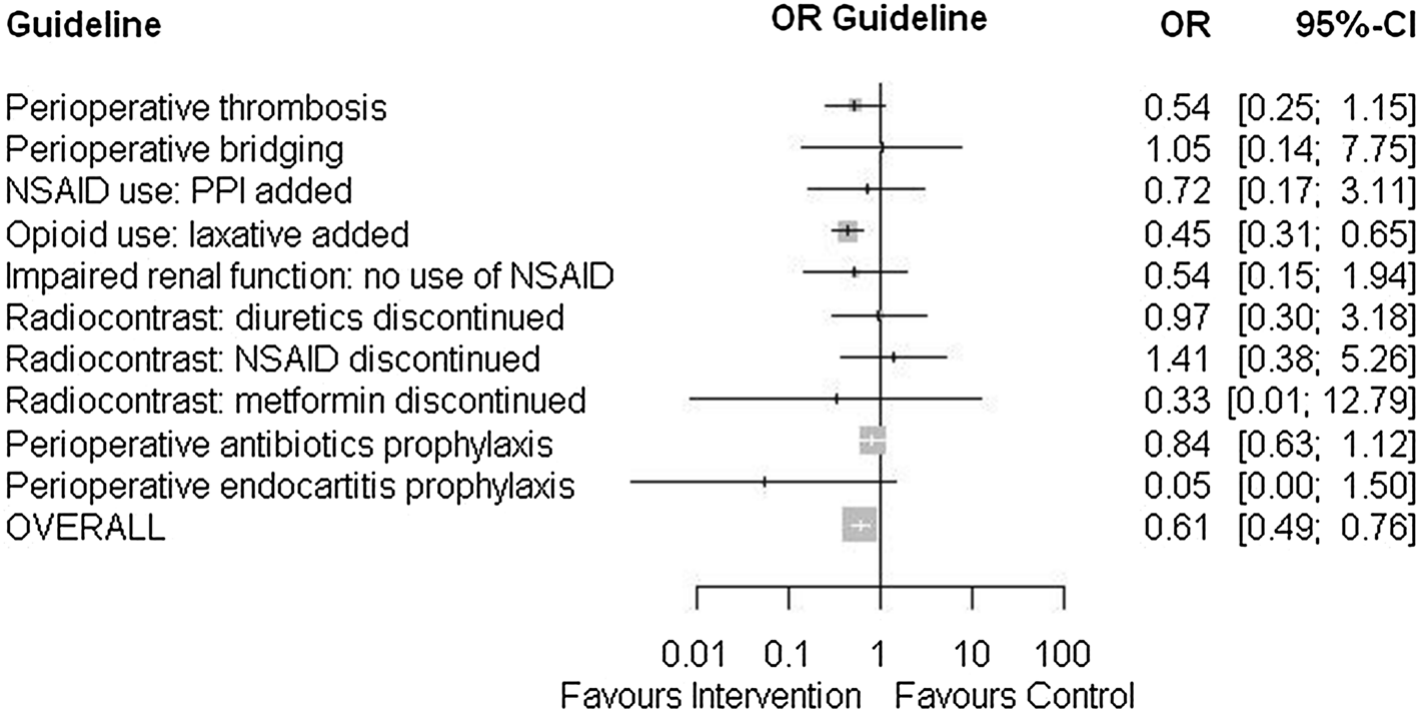
Forest plot of non-adherence of prescribers to pharmacotherapeutic measures based on prevailing guidelines

Table 3 shows the proportions of admissions of patients in which the physician did not adhere to the guidelines in the usual care period and in the intervention period, respectively. In 1089 admissions of 1069 patients in the usual care period and in 886 admissions of 864 patients in the intervention period, one or more included guidelines were applicable. Figure 1 shows a forest plot in which the odds ratios for non-adherence are presented.

**Table 3:** Non-adherence of prescribers to pharmacotherapeutic measures based on prevailing guidelines

	Usual care period (n = 1435)	Intervention period (n = 1195)	Odds ratios and confidence intervals	
	Non-adherence	Non-adherence	OR	95% CI
1. Perioperative thrombosis prophylaxis if indicated?	22/590 (3.7%)	10/490 (2.0%)	0.54	0.25–1.15
2. Perioperative bridging of antithrombotics if indicated?	2/48 (4.2%)	2/46 (4.3%)	1.05	0.14–7.75
3. In case of NSAID use, ppi added if indicated?	5/101 (5.0%)	3/83 (3.6%)	0.72	0.17–3.11
4. In case of opioid use, laxative added if indicated?	154/296 (52%)	62/190 (32.6%)	0.45^b^	0.31–0.65
5. In case of impaired renal function (MDRD < 30), no use of NSAID?	8/50 (16.0%)	4/40 (10.0%)	0.54	0.15–1.94
6. In case of radiocontrast, diuretics discontinued?	16/23 (69.6%)	20/29 (69.0%)	0.97	0.30–3.18
7. In case of radiocontrast, NSAID discontinued?	17/25 (68.0%)	15/20 (75.0%)	1.41	0.38–5.26
8. In case of radiocontrast and MDRD < 60, metformin discontinued?	2/3 (66.7%)	2/2 (100.0%)	0.33	0.01-12.79
9. Perioperative antibiotics prophylaxis, if indicated?	136/832 (16.3%)	93/661 (14.1%)	0.84	0.63–1.12
10. Perioperative endocarditis prophylaxis, if indicated?	6/8 (75%)	0/3 (0%)	0.05	0.00–1.50
Overall non-adherence	332/1089 (30.5%)	193/886 (21.8%)	0.64^b^	0.52–0.78
			0.61^a,b^	0.49–0.76

^a^ OR, adjusted for confounders

^b^ Statistical significant

A significantly lower proportion of admissions of patients in which the physician did not adhere to the prevailing guidelines occurred in the intervention period (21.8% (193/886) compared to the usual care period [30.5% (332/1089)] (*p* < 0.05). The odds ratio (OR) was 0.64 (95% CI 0.52–0.78).

After correction for possible confounders, the adjusted OR was 0.61 (95% CI 0.49–0.76).

When the analysis was conducted with protocol non-adherence as denominator (total number of times that a guideline had to be followed by a prescriber) instead of admissions, results were comparable. [13.5% (211/1564) in the intervention period compared to 18.6% (368/1976) in the usual care period (*p* ≤ 0.05)]. The odds ratio (OR) was 0.68 (95% CI 0.57–0.82).

## Discussion

Our study shows that education and support of the prescribing physician with respect to high-risk patients in surgical departments can lead to reduced pharmacotherapeutic guideline non-adherence among prescribing physicians. Achieved effects were obtained on top of the effect of other measures as CPOE/CDS system and clinical rules, which were part of usual care.

Earlier studies describe interventions that aim to improve guideline adherence in the hospital. Hogli et al. describe an intervention study, reporting distribution of a recently published pocket version of the national guideline. This led to a substantial increase in prescribing of appropriate empirical antibiotics from 61.7 to 83.8% [20]. Schouten et al. implemented a multifaceted guideline-implementation strategy, considering the barriers of implementation of guidelines. They found an increase of the rate of guideline adherence of antibiotic prescription from 50.3 to 64.3% [19].

In the two hospitals that participated in this study, the guidelines targeted in our study had already been implemented. Nevertheless, we found that in nearly one third of cases physicians were non-adherent to the local hospital guidelines. This is in line with earlier research on this subject. Van den Bemt et al. found that the proportion of admissions not compliant with guidelines on gastric protection in case of use of NSAID in hospitalized surgical patients was 46.6% [16]. Drenth et al. found an adherence of 53.9% with a dosing guideline in patients with impaired renal function at hospital discharge [15]. Schilp et al. studied adherence to the guideline concerning identification and hydration of high-risk patients for contrast-induced nephropathy in different hospitals and found that only two third of the high-risk patients were hydrated before contrast administration [33]. Huijts et al. reported proportions of patients receiving guideline-adherent antibiotics for community acquired pneumonia from 30.5 to 62.9% [34].

Educating prescribers is a measure that can be taken on top of other measures to improve guideline adherence. Educating prescribers is only effective if it is a part of a multifaceted intervention [35]. Novel of the P-REVIEW education program is the combination with a weekly visit by the hospital pharmacist, who audited and improved guideline adherence. We aimed to boost the effect of the education program by the weekly visits of the hospital pharmacist. We previously showed that these weekly visits were feasible and also efficient. We hypothesized that the support by education of the prescriber by the hospital pharmacist in a more pro-active role is more efficient and effective than the traditional retrospective role of the hospital pharmacist in medication surveillance [22]. In this study we show that this intervention can lead to a significant decrease in non-adherence of guidelines, although there may be still room for improvement.

The study has several strengths.

The study was performed in two representative general teaching hospitals. The intervention was easily implemented, since both education and weekly visits were performed by health care providers already active in the hospital and the intervention did not lead to additional costs.

The intervention combines different strategies. These strategies address different factors that have been described to impair quality of care and can influence guideline adherence [17].

By educating the prescriber in the hospital and teaching the prescriber during medication safety consultation, the knowledge and skills needed to adhere to guidelines will improve. Also the attitude towards guidelines in general and motivation to adhere may improve. This study however, didn’t collect qualitative data from the prescribers to support this hypothesis, and this would be useful to integrate in future research.

These weekly visits of the hospital pharmacist can be considered as a continuous form of workplace-based education. This addresses the problem that a short-term education programme has been found to have only a transient effect on the frequency of prescribing errors [36].

There are some limitations as well. We selected ten relevant pharmacotherapeutic measures derived from several guidelines. This selection was not complete, and our results may not be generalisable to other or all guidelines. Also, the study was performed in two hospitals, which might limit the external validity of the study.

This study was not a randomised controlled study, but was performed in a before after design, introducing the possibility of confounding. Therefore, to adjust for confounding, we performed a multivariable logistic regression analysis. Yet, we may not have identified all potential confounders.

We defined the outcome measure as the proportion of the admissions of patients in which the physician did not adhere to one or more of the guidelines. We assumed that in every single admission a physician has to follow all guidelines. When a patient is readmitted, the treating physician is often different and guidelines can be different from an early admission of the patient. On the other hand adherence on guidelines in an early admission can lead to (not intended) adherence in a readmission.

For each guideline we only included these cases in which there was no possible discussion on adherence (Table 1). By specifying the cases, we minimized the possibility of intended non-adherence by the physician.

The P-REVIEW study describes 106 admissions with one or more clinically relevant, potentially preventable, drug-related events in the usual care period and 73 in the intervention period [22]. These drug-related events are divided into different types of events, such as haemorrhage, thrombosis, renal failure related events, central nervous systems events, faecal impaction, hypoventilation and a group of unclassifiable events [22]. We noted that 25 of the 106 events in the usual care period and 15 of the 73 events in the intervention period relate to the studied guidelines and could possibly have been prevented in case of better guideline adherence. That means that only a modest part of the positive effect on these events can be related to an improvement in adherence of the studied guidelines.

This suggests that improving guideline adherence will have only limited effect and it may not be necessary to pursue 100% adherence. It may be better not to focus on guideline adherence or implementation of clinical rules alone, but on a comprehensive medication review of high risk patients, in which a check on adherence of guidelines and clinical rules is integrated.

Given the limited resources in healthcare, we think this could be an important question for future research.

## Conclusions

In summary, this study shows that education and support of the prescribing physician with respect to high-risk patients in surgical departments leads to an improvement of guideline adherence among prescribing physicians.
